# Efficacy of hyaluronic acid 0.3%, cyanocobalamin, electrolytes, and P-Plus in menopause patients with moderate dry eye disease

**DOI:** 10.1007/s00417-021-05415-6

**Published:** 2021-09-25

**Authors:** Concepción De-Hita-Cantalejo, María Carmen Sánchez-González, Carmen Silva-Viguera, Marta C. García-Romera, Ricardo Feria-Mantero, José-María Sánchez-González

**Affiliations:** Department of Physics of Condensed Matter, Optics Area, Pharmacy Faculty, University of Seville, Reina Mercedes Street, 41012 Seville, Spain

**Keywords:** Hyaluronic acid, Vitamin B12, Dry eye disease, Menopause, Artificial tears

## Abstract

**Purpose:**

To study the treatment efficacy of hyaluronic acid 0.3%, cyanocobalamin (vitamin B12), electrolytes, and P-Plus in menopausal patients with moderate dry eye disease.

**Methods:**

Thirty female patients of mean age 53.06 ± 5.20 years (45–65) were enrolled in this prospective longitudinal study. Meibomian gland loss assessment was determined using a scale with four levels. The Ocular Surface Disease Index (OSDI) questionnaire, phenol red thread (PRT) test, and tear film break-up time (TFBUT) were also completed by the patients. Tear eye drops were formulated with 0.3 g of sodium hyaluronate, P-Plus ™, vitamin B12, sodium chloride, potassium chloride, calcium chloride, magnesium chloride, and SCO® (stabilized complex oxychloride). After 30 days, the patients were re-evaluated.

**Results:**

The mean meibomian gland loss percentage was 37.97 ± 19.02 % (7.20 to 88.30%). Before treatment, the OSDI was 22.53 ± 14.03 score points (6.25 to 77.08). Posterior OSDI decreased to 16.26 ± 13.69 score points (0.00 to 70.83) (*W* = 58.00, *P* < 0.01). Before treatment, PRT was 10.31 ± 4.48 mm (4.00 to 21.00). Posterior PRT increased to 15.41 ± 6.27 mm (4.00 to 21.00) (*W* = 1520.50, *P* < 0.01). Before treatment, TFBUT was 6.23 ± 1.75 s (3.00 to 9.00). The posterior TFBUT increased to 8.10 ± 2.06 s (4.00 to 14.00) (*W*= 1382.50, *P* < 0.01).

**Conclusion:**

The hyaluronic acid 0.3% and vitamin B12 eye drops effectively decreased dry eye symptoms in menopausal women and improved tear stability and volume.

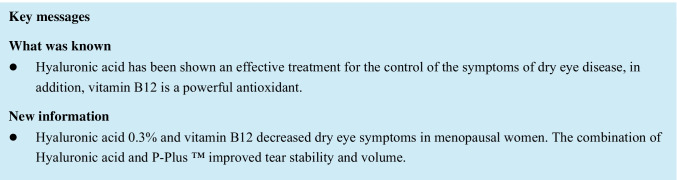

## Introduction

Menopause is associated with the physiological interruption of the ovarian hormonal secretion of estrogen and progesterone. Consequently, pathological processes can develop in different locations, such as the urogenital system [1], cardiovascular system [2], and bones [3]. Additionally, some ocular structures may be altered. As a consequence of physical and psychological deterioration, the quality of life of menopausal women may be reduced [4]. Dry eye syndrome is a disease characterized by the pathological restructuring of tears and lesions on the corneal surface, which result in symptoms, such as visual discomfort and disturbance, burning, itching, redness, pain, and ocular fatigue, without uniform diagnostic criteria [5]. Maintaining physiological levels of hormones, such as estrogens, is essential to obtain stable tear quality. Estrogen and androgen levels affect the three components of the tear film, the aqueous layer, lipids, and mucin. During menopause, there is a decrease in the level of many sex hormones that subsequently results in dry eye disease (DED) [6].

Further, a positive correlation has been demonstrated between age and dry eye syndrome symptoms [7], and a synergistic effect between physiological factors linked to age and hormonal changes in menopause is possible. The prevalence of ocular surface disease symptoms in menopausal women was 64% (45–79 years), with a mean age of 49.45 ± 4.02 years at menopause [8]. Tear acts as the substitute to increase humidity at the ocular surface and improve lubrication, consequently reducing symptoms in most cases [9]. Currently, there are several strategies for the treatment of dry eyes, whereby autologous serum [10], surgical interventions [11] or combinations of lubrication and anti-inflammatory agents [12] have been used. In many cases, nutritional supplementation [13], such as that with omega-3 and omega-6 fatty acids, and temporary occlusion of the dry eye [14] are considered reasonable recommendations. The current understanding of the ocular surface changes in patients with dry eye syndrome results from hyperosmolarity of the tear film, inflammatory processes, and oxidative stress. We studied an artificial tear therapy formulated with hyaluronic acid, a compound widely used as an ocular lubricant in isolation or in combination with other substances [15]. P-Plus™ provides viscosity and adherence to the tears, and cyanocobalamin (vitamin B12) has antioxidant and anti-inflammatory properties [16].

The primary purpose of this study was to determine the treatment efficacy of 0.3% hyaluronic acid, cyanocobalamin, electrolytes, and P-Plus in menopausal patients with moderate dry eye disease.

## Methods

### Design and subjects

Thirty female patients were enrolled in this prospective longitudinal study that was developed in the optometry cabinets of the Faculty of Pharmacy at the University of Seville (Spain) between January 2019 and March 2019. This study was approved by the Ethics Committee of Andalusia and conducted following the principles of the Helsinki Declaration.

The inclusion criteria were as follows: (1) women in menopause or perimenopause; (2) age between 45 and 65 years; (3) a break-up time of <10 s; (4) completion of all examinations; and (5) a full understanding of the meaning of the study and a signed informed consent form before the start of measurements. The exclusion criteria were as follows: (1) any previous eye surgery, (2) any systemic diseases, (3) any pharmacological treatment (including hormone replacement therapy or meibomian gland drain instruments as lipiflow), and (4) contact lens wearers. Out of a total of 36 women who met the inclusion but not the exclusion criteria, two decided not to take rejection tests, two others did not complete the full study, and two others did not show up.

### Material

Non-contact infrared meibography (CSO Non-mydriatic Fundus Camera Cobra HD, Italy) was used for the meibomian gland (MG) structure and health analysis using Ana Eyes software (Essilor Instruments, USA). A scale determined MG loss assessment (meiboscore) with four levels; grade 0 exhibited no loss, and grades 1, 2, and 3 exhibited < 33%, 33–66%, and > 66% loss [17, 18]. Patients also completed the Ocular Surface Disease Index (OSDI) questionnaire. The questionnaire was used to assess DED symptomatology perceived by the patient during the previous month. The questionnaire consisted of three blocks, each with four questions (12 issues in total). The total OSDI score was obtained from the total sum of the value answered to each question, measured from 0 to 4, multiplied by a fixed value of 25, and divided by the number of questions answered, resulting in a percentage that measured the probability of presenting dry eye between 0 and 50% [19, 20]. The phenol red thread test (PRT) (Tianjin Jingming New Technological Development Co., Ltd., China) was used to measure tear film secretion. It consisted of a cotton thread placement impregnated with phenol red onto the temporal one-third of the lower eyelid. The thread is yellow when dry and changes to red when moistened by tears because it is sensitive to pH. After 15 s, the thread was removed, and the period when it changed to red was measured [19, 20]. The procedure was performed first in the right eye followed by the left eye. The tear film quality was measured by the tear film break-up time test (TBUT) using a fluorescein strip (Bio 62 Glo ContaCare Ophthalmic & Diagnostics, Gujarat, India) impregnated with saline solution drops and a slit lamp (TOPCON SL-6E, Japan). The strip was used to dye the upper bulbar conjunctiva. The patient was instructed to blink naturally three times and stop blinking until instructed, and the tear film was observed with cobalt blue illumination of a slit lamp. The interval of time in seconds between the last complete blink and the appearance of the first break in the tear film was reported as the tear film break-up time (TFBUT)[19, 20]. The average value of three consecutive measurements was used for analysis.

Tear eyedrops (VISIONLUX^®^ PLUS, Novax^®^ Pharma, Monaco, France) were formulated with 0.3 g of sodium hyaluronate, P-plus^TM^, vitamin B12, sodium chloride, potassium chloride, calcium chloride, magnesium chloride, and SCO® (stabilized complex oxychloride). This preservative when exposed to sunlight breaks down into natural components that are not toxic to the ocular surface. All components were dissolved in a buffered isotonic solution. Hyaluronate sodium 0.3% was obtained by fermentation and was not of animal origin. Sodium hyaluronate activity was optimized with the synergistic action of P-plus™. P-Plus™ is a water-soluble polymer with phylogenic properties and lubricants. The hyaluronate association of sodium and P-Plus™ not only increased the viscosity of the solution but also improved its mucoadhesive properties. The presence of electrolytes (Cl–, Na+, K+, Ca++, and Mg++) essential for the cellular biochemical process helped maintain the eye surface in good physiological conditions. Vitamin B12 has antioxidant properties, thereby protecting the eye surface from damage induced by reactive oxygen species (ROS). Therefore, it was an essential factor in maintaining a healthy eye surface. It was packaged in a 10-ml multidose bottle. Laboratory recommended dosage was twice a day.

### Examinations procedure

The study consisted of two phases. In the first phase, patients were selected, and a biomicroscope examination was performed to assess their suitability for inclusion in the study. The patients completed the OSDI questionnaire. Tear volume was evaluated using the PRT test and tear quality with the TBUT test using fluorescein and the cobalt blue light of the slit lamp. MG loss assessment was evaluated by meibography (meiboscore). After a zero time of 15 days without tear treatment, patients were instructed on the correct instillation technique and the dose for tear eye drops (twice instillation per day). After 30 days, in the second phase, patients were re-evaluated and tested using the OSDI, PRT, and TFBUT. To avoid the subjective bias of the researcher, the results of the measurements obtained during the second visit were recorded in a different document from that of the first measurements. Finally, the data collected in both phases were compared to obtain the results of the study. All examinations were conducted by an experienced optometrist, at the same location, under the same time and humidity conditions, and using the same instrumentation.

### Statistical analysis

The sample size was assessed with the GRANMO® calculator (Institut Municipal d’Investigació Mèdica, Barcelona, Spain, Version 7.12). Accepting an alpha risk of 12:05 and a beta risk of 0.2 in a two-sided test, 21 subjects must recognize a statistically significant difference consisting of an initial proportion of 0.8 and a final proportion of 0.2. A drop-out rate of 10% was anticipated. Statistical analysis was performed using SPSS statistical software (version 26.0, IBM Corp, Armonk, New York, USA). The normality of the data distribution was assessed using the Shapiro-Wilk test. Descriptive analysis was performed using the mean ± SD (standard deviation; variability value). Differences between previous and posterior treatment variables were assessed using the Wilcoxon test. Effect size calculation was assessed within D of the Cohen test. Qualitative differences were assessed using the chi-squared test or McNemar test. The correlation study was assessed using the Spearman Rho test. The level of significance was set at 95% (*P*-value < 0.05).

## Results

**Figure 1:**
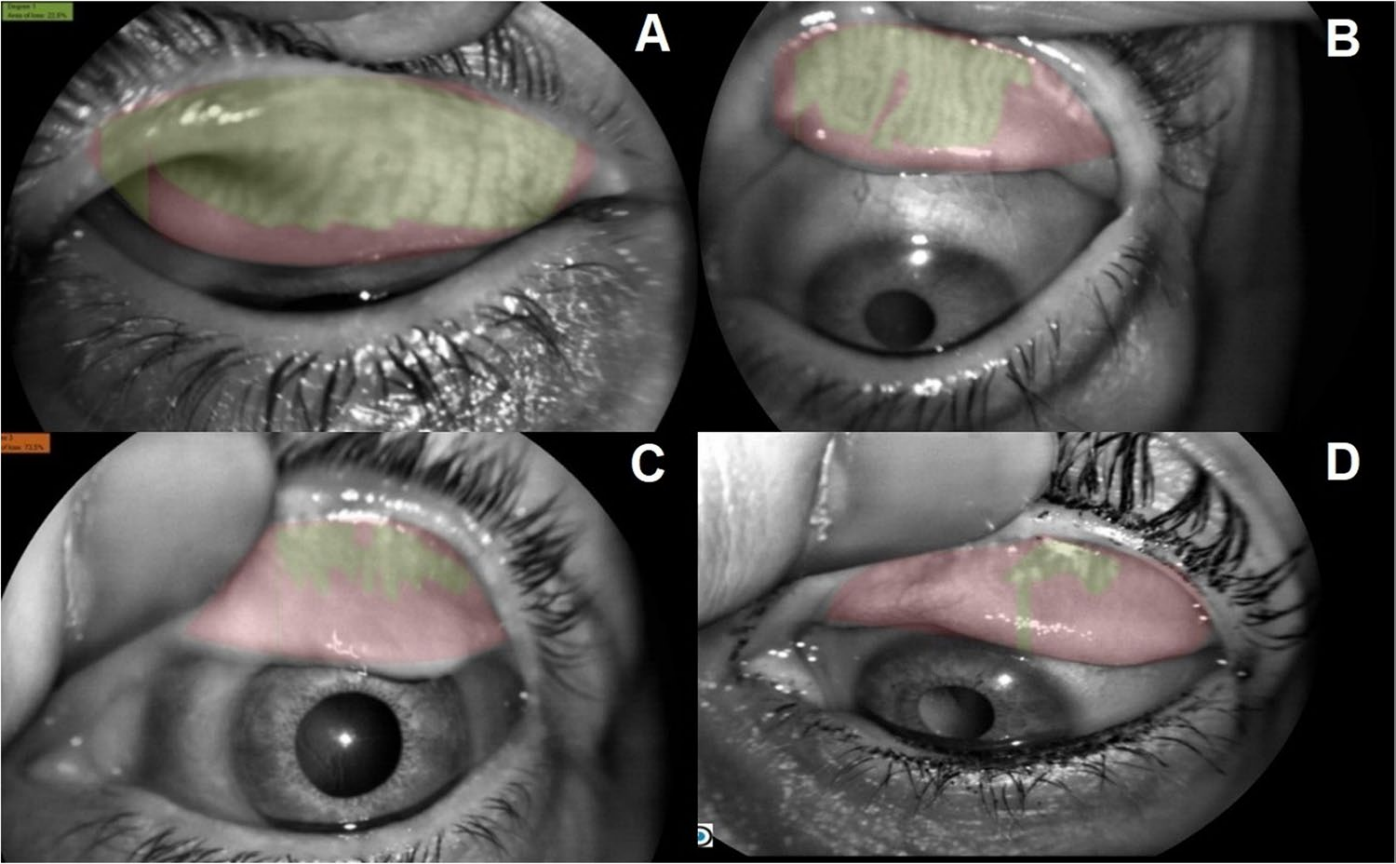
Meibomian gland dysfunction (MGD) models according to the meibomian score rank described by Arita et al. [17, 18], which was based on the percentage of meibomian gland loss determined using image processing software. All captions were from an upper lid. **A** Meibomian gland loss of 22.8%, achieving grade 1 of MGD. **B** Meibomian gland loss of 45.8%, achieving grade 2 of MGD. **C** Meibomian gland loss of 73.5%, achieving grade 3 of MGD. **D** Meibomian gland loss of 87.5%, achieving grade 3 of MGD

**Figure 2:**
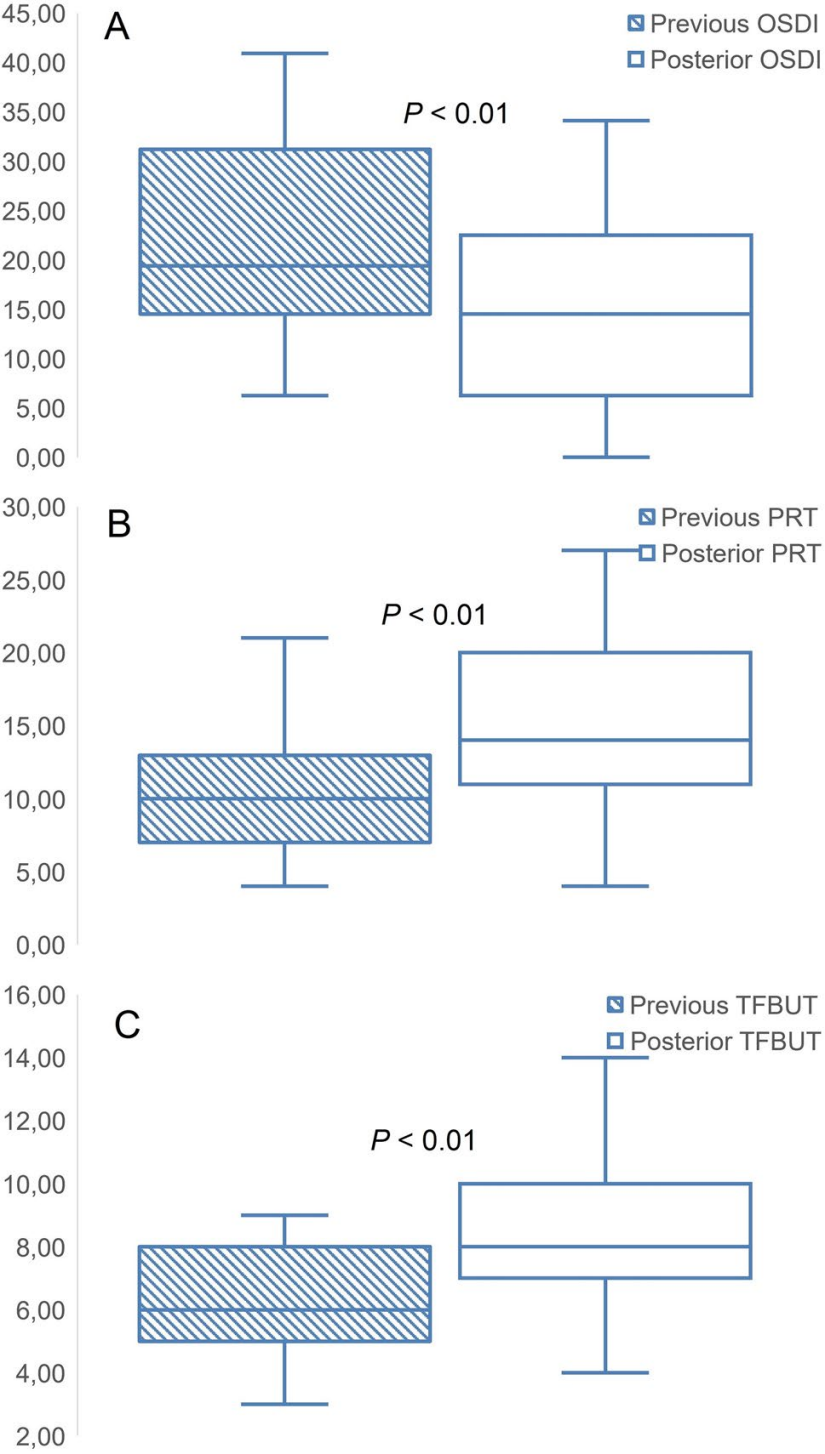
Box and plot graphs of longitudinal differences within three main variables studied. **A** Ocular Surface Disease Index (OSDI), measured in score points. **B** Phenol red test (PRT), measured in millimeters. **C** Tear film break up time (TFBUT), measured in s

Thirty women were enrolled in this longitudinal study. The mean age of the women included in the study was 53.06 ± 5.20 years (45–65). The mean MG loss percentage was 37.97 ± 19.02% (7.20 to 88.30), and the mean MG loss grade according to Arita et al. [17, 18], measured by the meibomian score rank, was 1.70 ± 0.67 (grade 1 to grade 3). Regarding the MG loss grade frequency, 25 patients achieved grade 1 (41.7%), 28 patients achieved grade 2 (46.7%), and only seven patients achieved grade 3 (11.7%). Four representative MG images of different degrees and percentages of MG loss are shown in Fig. 1. MG loss assessment was not performed after eyedrop treatment because it has been shown that artificial tears do not alter the MG pattern [21].According to the McNemar test (P<0.01). OSDI, PRT and TFBUT box and plot graph were presented in Fig. 2.

### OSDI

A quantitative analysis was performed before the treatment; the Ocular Surface Disease Index (OSDI) was 22.53 ± 14.03 score points (6.25 to 77.08). Posterior OSDI decreased to 16.26 ± 13.69 score points (0.00 to 70.83). Significant statistical differences occurred between the previous and posterior OSDI values (W = 58.00, *P* < 0.01). Positive differences (OSDI increased) were found only in four eyes, negative differences (OSDI decreased) in 52 eyes, and ties (same OSDI) only in four eyes. The effect size was 0.4523, and according to Cohen’s D results, it was a medium effect size. Spearman’s rho between the previous and posterior OSDI was 0.824 (*P* < 0.01).

Additionally, a qualitative analysis was performed. According to the Tear Film and Ocular Surface International Dry Eye Workshop (DEWS II) [19, 20], the OSDI test was classified into four ranks (normal < 13 score points, mild between 13 and 22, moderate between 23 and 32, and severe ≥ 33). In the previous OSDI, 14 eyes reached normal score points (23.3%), 26 eyes attained mild score points (43.3%), 12 eyes achieved moderate score points (20.0%), and only eight eyes achieved severe score points (13.3). In the posterior OSDI, 26 eyes reached normal score points (43.3%), 20 eyes attained mild score points (33.3%), 10 eyes achieved moderate score points (16.7%), and only four eyes achieved severe score points (6.7%). According to the chi-square test, this represented a statistically significant difference (*χ*^2^ = 47.37, *P* < 0.01).

### PRT

A quantitative analysis was performed before the treatment. The PRT was 10.31 ± 4.48 mm (4.00 to 21.00). Posterior PRT increased to 15.41 ± 6.27 mm (4.00 to 21.00). Significant statistical differences occurred between the previous and posterior PRT (*W* = 1520.50, *P* < 0.01). Positive differences (PRT increased) occurred in 48 eyes, negative differences (PRT decreased) in 10 eyes, and ties (same PRT) only in two eyes. The effect size was 0.9360, and according to Cohen’s D results, it was a considerable effect size. Spearman’s rho between previous and posterior OSDI was 0.295 (*P* < 0.05).

Additionally, a qualitative analysis was performed. According to DEWS II [19, 20], PRT test cut-off point was 10 mm. In the previous PRT, 26 eyes were under the cut-off point (43.3%), and 34 eyes were above the cut-off point (56.7%). Only eight eyes were under 10 mm (13.3%) in the posterior PRT, and 52 eyes were above 10 mm (86.7%). This represented a statistically significant difference, according to the McNemar test (*P* < 0.01).

### TFBUT

A quantitative analysis was performed before the treatment. TFBUT was 6.23 ± 1.75 s (3.00 to 9.00). The posterior TFBUT increased to 8.10 ± 2.06 s (4.00 to 14.00). Significant statistical differences were found between the previous and posterior TFBUT (*W* = 1382.50, *P* < 0.01). Positive differences (TFBUT increased) were found in 49 eyes, negative differences (TFBUT decreased) in four eyes, and ties (same TFBUT) in only seven eyes. The effect size was 0.9941, and according to Cohen’s D results, it was a considerable effect size. Spearman’s rho between previous and posterior TFBUT was 0.694 (*P* < 0.01).

Additionally, a qualitative analysis was performed. According to DEWS II [19, 20], TFBUT test cut-off point was 10 s. In the previous TFBUT, all eyes were under 10 s (100%). In the posterior TFBUT, only 41 eyes were under 10 s (68.3%), and 19 eyes were above 10 s (31.7%). This represented a statistically significant difference, according to the McNemar test (*P* < 0.01).

## Discussion

This prospective longitudinal study aimed to study the efficacy of eye drops with 0.3% hyaluronic acid and vitamin B12 in menopausal women after 1 month of use. Hyaluronic acid is considered an effective treatment for the control of the symptoms of DED [22–25]. On the other hand, vitamin B12 is a powerful antioxidant, observing that certain hyaluronic acid eye drops with vitamin B12 can reduce oxidative stress and improve dry eye symptoms [26]. MG assessment was performed on all patients, showing the dry eye symptoms characteristic of gland loss [27]. Additionally, some studies show dry eyes in women of this age. García-Alfaro et al. [8] conducted a study in perimenopausal and postmenopausal women to evaluate the symptoms of dry eye using the OSDI questionnaire, concluding that this symptomatology exists in both groups and may affect activities related to vision along with a decrease in quality of life. Similarly, Careba et al. [28] conducted a study with 66 postmenopausal women in which ocular dryness was evaluated using the OSDI, TBUT, and non-invasive TFBUT, in which they found that the group of women with symptoms of ocular dryness was related to data from the higher OSDI tests, shorter TBUTs, and non-invasive TFBUT. Additionally, various studies have considered this test reliable for the measurement of this symptomatology[29, 30].

According to the tests performed, our results indicated that all women exhibited improved dry eye symptoms after treatment (OSDI, PRT, and TFBUT). The decrease in OSDI in 52 eyes indicated patient satisfaction with the use of drops for relieving symptoms. Serrano-Morales et al. [31] conducted a study in menopausal women to demonstrate the efficacy of hyaluronic acid, concluding that after 2 months of treatment, the OSDI test score decreased, coinciding with the data from our study. In the PRT test, considering the cut-off point at 10 mm, 86.7% had more than 10 mm after the month of treatment, resulting in a higher result in 48 eyes. Saeed et al.[32] conducted a study of the efficacy of hyaluronic acid in dry eyes in which three measurements (baseline 4–8 weeks) of the Schirmer’s test were performed, showing how in each case there was an increase in the results, which corroborated our efficacy data using this test. Although Schirmer’s test was performed in this study, some studies equalize the efficacy of both tests for the diagnosis of tear quantity [33, 34].

In the TFBUT test, positive statistically significant differences were found in 49 eyes and the same result in only seven eyes. Cheo et al. [24] conducted a study comparing the efficacy of 0.1%, 0.18%, and 0.3% hyaluronic acid eye drops in mice and observed that the TBUT increased after 28 days of treatment. They concluded that drops with a concentration of 0.3% were more useful for the treatment of dry eye, thereby supporting our results and indicating that these drops are more effective than those with a lower concentration of hyaluronic acid. Therefore, it was concluded that vitamin B12 is an essential component of these artificial tears, with antioxidant properties that protect the ocular surface. Macri et al. [26] conducted a study with 0.15% hyaluronic acid eye drops and vitamin B12 to evaluate the effects on oxidative stress and dry eye and concluded that both were related and that the combined composition of the drops reduced stress and inflammation and improved all dry eye symptoms. Previously, we mentioned the greater efficacy of 0.3% hyaluronic acid present in our tears, and this along with the antioxidant effects of vitamin B12 confirmed the efficacy of the data from all the tests conducted.

Additionally, it should be noted that the tears used are composed of a water-soluble polymer, P-Plus ™, which, together with hyaluronic acid, tends to increase viscosity and improve mucoadhesive properties. Aragona et al. [35] conducted a study in which they analyzed the physicochemical properties of 18 drops with hyaluronic acid and with different polymers and concluded that tears in the presence of polymers increased viscosity were more beneficial for patients and increased the properties of hyaluronic acid in the treatment of dry eyes; composition of the drops used in our study demonstrated similar effects. Our study had some limitations. First, the sample consisted of 30 patients, and higher patient volume should include in future research. Additionally, the study was designed as a longitudinal one-arm study, and a longer follow-up is needed to confirm the achieved results.

In conclusion, 0.3% hyaluronic acid and vitamin B12 eye drops effectively decreased dry eye symptoms in menopausal women and improved tear stability and volume.

## Data Availability

Data are available from the corresponding author upon reasonable request.
